# Supplementary material for: Comparison of polypeptides that bind the transferrin receptor for targeting gold nanocarriers
Source: PLoS One. 2021 Jun 4;16(6):e0252341. doi: 10.1371/journal.pone.0252341 (PMC8177412; doi:10.1371/journal.pone.0252341)
Supplement: S1 Table — (DOCX) [file pone.0252341.s001.docx]

**S1 Table**

Specific fluorescence and concentrations of peptides in culture, net charge at pH 7 and proportion of hydrophobic residues

| **Peptide** | **Fluorescence**  **(Units/ μg)** | **Concentration**  **(μg/ml)** | **Charge** | **Hydrophobicity** |
| --- | --- | --- | --- | --- |
| Pep-1 | 24026 | 16.8 | -3 | 39% |
| Pep-2 | 33800 | 12.0 | -1 | 33% |
| Pep-10 | 14497 | 28.1 | -2 | 33% |
| Pep-R1 | 40723 | 10.0 | -3 | 37% |
| Pep-R2 | 71265 | 5.7 | -1 | 37% |
| Pep-L | 19221 | 21.2 | +1 | 57% |
| Dextran | 16420 | 24.8 |  |  |
