# Supplementary figures and images for: Comparison of polypeptides that bind the transferrin receptor for targeting gold nanocarriers
Source: PLoS One. 2021 Jun 4;16(6):e0252341. doi: 10.1371/journal.pone.0252341 (PMC8177412; doi:10.1371/journal.pone.0252341)

**Supplementary Fig.2 Dose dependency of endocytosis of Pep-10 and Pep-L by hCMEC/D3 cells**


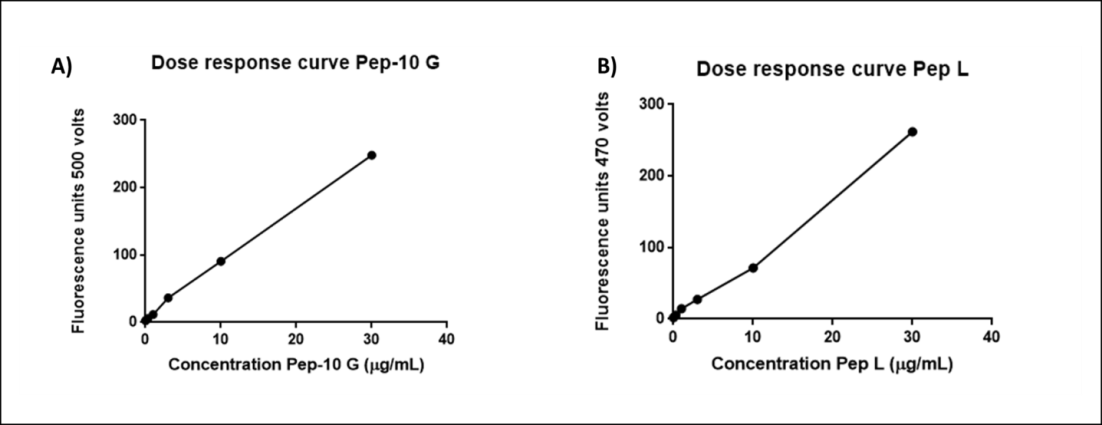

Supplement: S1 Fig — (DOCX) [file pone.0252341.s002.docx]
