# Supplementary material for: Comparison of polypeptides that bind the transferrin receptor for targeting gold nanocarriers
Source: PLoS One. 2021 Jun 4;16(6):e0252341. doi: 10.1371/journal.pone.0252341 (PMC8177412; doi:10.1371/journal.pone.0252341)
Supplement: S2 Fig — (DOCX) [file pone.0252341.s003.docx]

**Supplementary Fig.3 Expression of TfR on hCMEC/D3 cells measured by cell-surface ELISA**
